# Post-traumatic growth in patients and caregivers after a primary brain tumor diagnosis

**DOI:** 10.1007/s11060-026-05454-1

**Published:** 2026-02-11

**Authors:** Ashlee R. Loughan, Sarah Ellen Braun, Autumn Lanoye, Kelcie Willis, Max Hernand

**Affiliations:** 1 School of Medicine, Virginia Commonwealth University, Richmond, VA USA; 2 Massey Comprehensive Cancer Center, Richmond, VA USA; 3 Dana Farber Cancer Center, Boston, MA USA; 4 Department of Psychology, Virginia Commonwealth University, Richmond, VA USA; 5 Division of Neuro-Oncology, VCU Massey Cancer Center, Department of Neurosurgery, School of Medicine, Virginia Commonwealth University, 1201 East Marshall St., Richmond, VA 23298-0037 USA

**Keywords:** Post traumatic growth, Neuro-oncology, Patients, Caregivers, Wellbeing

## Abstract

**Purpose:**

Individuals with primary brain tumors and their caregivers experience substantial psychological distress, yet positive psychological outcomes, such as post-traumatic growth (PTG), remain understudied. This study aimed to assess the prevalence and profile of PTG among primary brain tumor patients and caregivers, examine associations with demographic, medical, and psychological variables, and inform future neuro-oncology interventions.

**Methods:**

In this prospective cross-sectional study, 141 participants (96 patients, 45 caregivers) completed validated surveys assessing PTG, depression, anxiety, and death anxiety. Participants were recruited via online neuro-oncology support and advocacy groups. PTG was measured using the Post-Traumatic Growth Inventory (PTGI). PTG profiles were examined using descriptives and associations between PTG and patient/caregiver characteristics, tumor-related medical variables, and psychological distress were examined using t-tests, ANOVAs, and Pearson correlations.

**Results:**

70% of patients and 48.8% of caregivers reported moderate-to-high PTG. Appreciation of Life was the most frequently endorsed subscale in both groups. Patients with high-grade tumors exhibited significantly higher PTG than those with low-grade tumors; caregivers of patients with left-hemisphere tumors trended toward higher PTG. PTG was positively associated with death anxiety in patients but unrelated to depression or generalized anxiety in either group.

**Conclusion:**

Despite significant distress, many patients and caregivers experience PTG following a brain tumor diagnosis, particularly in appreciating life. These findings highlight the potential for psychosocial and palliative interventions to harness PTG through meaning-making and existential engagement.

## Introduction

Individuals diagnosed with primary brain tumors frequently report profound disruptions to their overall quality of life, particularly marked by elevated levels of emotional distress. Among survivors, there is a high prevalence of depressive symptoms (up to 95%) [1–3], anxiety symptoms (up to 63%) [4], and existential distress (up to 50%) [5, 6]. Importantly, these psychological burdens are not limited to patients alone. Caregivers of individuals with brain tumors also report significant emotional strain, often surpassing the distress levels experienced by the patients themselves [7–9]. However, a critical question remains: Are distress and suffering the only psychological responses in the context of brain tumor survivorship and caregiving, or is there a broader spectrum of emotional experiences yet to be fully explored?

Post-Traumatic Growth (PTG) refers to the positive psychological transformation that can emerge in the aftermath of traumatic life events [10–12], such as the diagnosis of a life-limiting, aggressive brain tumor in oneself or a loved one. This growth may manifest as heightened resilience, enhanced coping strategies, and a deeper appreciation for life. PTG is generally understood to consist of five subdomains: relations with others (e.g., improved family functioning, more open communication), new possibilities (e.g., development of new interests/hobbies, greater likelihood to attempt to change one’s circumstances), personal strength (e.g., sense of self-reliance), spiritual changes (e.g., stronger religious faith), and appreciation of life (e.g., better understanding of priorities and values). Importantly, PTG does not imply the absence of psychological distress. Rather, it represents emotional development and skillful coping that surpasses one’s pre-trauma baseline [11, 13]. When individuals encounter experiences that fundamentally disrupt their assumptions about the world’s predictability, controllability, and benevolence, they are often compelled to reconstruct a new existential framework—one that helps them make meaning of their circumstances. A brain tumor diagnosis, with its profound psychological and existential implications, presents a unique context in which both patients and caregivers may cultivate PTG amid significant adversity; however, investigations of PTG in this population remain scarce [14]. 

Higher levels of PTG have been reported by cancer survivors when compared to individuals who experienced other adverse life events [15], specifically within the domains of personal strength and spiritual development [16]. Moreover, among those with cancer, PTG has the potential to mediate the relationship between psychosocial stressors (e.g. traumatic stress, loss of meaning, socioeconomic vulnerability) and life quality [15, 17]. Recently, our team conducted a scoping review of PTG to determine the representation of primary brain tumor inclusion in the literature, summarize the findings to date, and identify key gaps in the literature for future investigation [14]. We found that patients with primary brain tumors were adequately represented in the current PTG literature (1.19%) relative to the prevalence of primary brain tumors among all cancers (1.4%); nevertheless, relatively few of the identified articles (*n* = 5) focused exclusively on the brain tumor experience, and instead, presented results from mixed cancer samples. The studies that were specific to neuro-oncology, solely focused on low-grade glioma samples, highlighting a critical gap in understanding PTG among those with high-grade tumors. Moreover, none of these investigations examined PTG in caregivers. Given the life-altering and existential reality of a brain tumor diagnosis, further investigations of PTG in neuro-oncology – in both patient and caregiving samples – are therefore warranted.

To address the identified gaps, the present study aimed to: (1) assess the prevalence and specific profile of PTG among patients with primary brain tumors and caregivers of those with primary brain tumors—specifically, across tumor grade diagnostic classifications; (2) explore associations between PTG and relevant demographic, medical, and psychological variables; and (3) provide evidence-based recommendations for future research, with a particular focus on informing the development of psychological and palliative care interventions in neuro-oncology.

## Methods

### Participants and procedure

In this prospective cross-sectional study, participants diagnosed with a primary brain tumor and caregivers of those diagnosed with a primary brain tumor completed surveys that assessed: self-reported demographic and medical characteristics, PTG, and psychological distress (depressive symptoms, generalized anxiety, death anxiety). All recruitment and study procedures were conducted virtually. Patients and caregivers were recruited from neuro-oncology social media support groups (e.g., Facebook, Twitter). Many of these social media groups require moderator verification prior to granting membership or posting privileges, which typically involves answering questions related to one’s brain tumor diagnosis, treatment history, or one’s connection to the brain tumor community. If eligible, participants completed all surveys virtually via REDCap [18] on a personal electronic device. Recruitment and data collection occurred from June 2020 – March 2021. Ethical approval was granted from the institutional review board (HM20013477) and informed consent was obtained from all study participants prior to any data collection. Inclusion criteria for patients were: (1) age 18 or older, (2) self-reported primary brain tumor diagnosis, and (3) self-reported literacy in English. Inclusion criteria for caregivers were: (1) age 18 or older, (2) self-reported caregiver of a patient with primary brain tumor, and (3) self-reported literacy in English. Caregiver enrollment was not dependent on patient enrollment and vice versa. Enrollment included 194 participants (129 patients and 65 caregivers). Fifty-three participants had incomplete data, thus a total of 141 participants (96 patients and 45 caregivers) were included in the final sample. A total of 14 patients and 14 caregivers enrolled as dyads, all other patients and caregivers represent non-dyadic data.

### Measures

#### Demographic and medical characteristics

Patients and caregivers were both asked to report sociodemographic information (e.g., age, gender identity, race/ethnicity, education, relationship status, employment status), neuro-oncology diagnostics (e.g., tumor type, tumor grade, tumor hemisphere, time since diagnosis), and brain tumor treatment history (e.g., surgical resection, cranial irradiation, chemotherapy).

#### Post-Traumatic Growth Inventory (PTGI) [12]

The PTGI is a 21-item validated self-report measure designed to assess the extent to which individuals coping with the aftermath of trauma are successful in reconstructing or strengthening their perceptions of self, others, and the meaning of events. For this study, participants were instructed to anchor PTG responses to their brain tumor diagnosis. Items are summed to yield a total score ranging from 0 to 105, with higher scores indicative of greater growth. There are five subscale scores: (1) relations with others (7 items); (2) new possibilities (5 items); (3) personal strengths (4 items); (4) spiritual changes (2 items); and (5) appreciation of life (3 items). For PTGI total score, scores of 45 and below represent none-to-low PTG levels, whereas scores of 46 and above represent moderate-to-high PTG levels [19, 20]. For PTGI subscales, calculated by averaging associated items, moderate-to-high was defined by a mean score *≥* 3 [21]. Cronbach’s alpha was 0.92 in patients and 0.95 in caregivers in the current study.

#### Patient Health Questionnaire-9 (PHQ-9) [22]

The PHQ-9 is a 9-item validated self-report measure designed for use in both clinical and research settings to assess severity of depressive symptoms. Possible total scores range from 0 to 36, with higher scores reflecting greater symptom severity. A score of 10 or higher has been established as a cut-point to indicate clinically significant symptoms of depression. Furthermore, scores of 5, 10, 15, and 20 indicate mild, moderate, moderately severe, and severe levels of depression, respectively. Cronbach’s alpha was 0.89 in both patients and caregivers in the current study.

#### General Anxiety Disorder-7 (GAD-7)[23]

The GAD-7 is a 7-item validated self-report measure designed to assess severity of generalized anxiety symptoms in both clinical and research settings. Items are summed to yield a total score ranging from 0 to 21, with higher scores indicative of greater symptom severity. Scores of 5, 10, and 15 correspond to mild, moderate, and severe levels of generalized anxiety, respectively. Cronbach’s alpha was 0.93 for patients and 0.92 for caregivers in the current study.

#### Death and Dying Distress Scale (DADDS)[24]

The DADDS is a 15-item validated self-report measure designed to assess severity of death anxiety, specifically in oncology populations. Items are summed to yield a total score ranging from 0 to 75, with higher scores indicative of greater death anxiety. Previous studies report that scores of 0–24 indicate low death anxiety, 25–46 indicate moderate death anxiety, and 47–75 indicate severe death anxiety. Cronbach’s alpha was 0.95 for patients and 0.92 for caregivers in the current study.

### Data analytic plan

Analyses were conducted in SPSS29. Data were cleaned and assumptions of planned analyses were checked. Mean subscale imputation was used for any missing data (0.3%). Statistical significance was tested at *p* < 0.05, and trending effects were defined as 0.05 > *p* < 0.10. To determine the prevalence of PTG in both patients and caregivers, descriptive analysis of the PTGI total score and subscales were completed. In order to examine the associations between PTG and patient and caregiver demographics, patient tumor-related medical characteristics, and psychological distress, we utilized Pearson correlations for continuous variables (i.e., age, time since diagnosis, generalized anxiety, depression, death anxiety), independent samples t-tests for comparison of dichotomous groups (i.e., PTG profile [patients v. caregiver], gender [male v. female], tumor grade [low v. high], relationship to patient [spouse/partner v. other]), and one-way analyses of variance for categorical variables with more than two levels (i.e., tumor type [glioblastoma v. astrocytoma, v. oligodendroglioma, v. meningioma], tumor hemisphere [left v. right, v. bilateral]). Due to the homogeneity of the patient and caregiver samples, we did not test associations between PTG and race/ethnicity.

## Results

Patient and caregiver demographics, patient medical variables, and psychological profiles are presented in Table 1.

**Table 1:** Demographics and medical variables

	Patients *n* = 96	Caregivers *n* = 45
**Demographic Variables**		
**Age (mean, range)**		
	46.63 years (23–73)	50.33 years (23–77)
**Gender (n**,**%)**		
Female	33 (34.4%)	34 (75.6%)
Male	62 (64.6%)	11 (24.4%)
Transgender/Nonbinary	1 (1.0%)	0 (0.0%)
**Race/Ethnicity (n**,** %)**		
African American/Black	1 (1.0%)	1 (2.2%)
Caucasian/White	88 (91.7%)	42 (93.3%)
Asian	3 (3.1%)	0 (0.0%)
Hispanic or Latino	2 (2.1%)	1 (2.2%)
Native Hawaiian or Pacific Islander	1 (1.0%)	0 (0.0%)
Other	1 (1.0%)	1 (2.2%)
**Relationship to Patient (n**,** %)**		
Spouse / Partner	--	29 (64.4%)
Family Member	--	15 (33.3%)
Friend	--	1 (2.2%)
**Medical Variables**		
**Tumor Type (n**,** %)**		
Glioblastoma	18 (18.8%)	21 (46.7%)
Astrocytoma	20 (20.8%)	7 (15.6%)
Oligodendroglioma	26 (27.1%)	8 (17.8%)
Meningioma	19 (19.8%)	4 (8.9%)
Other	13 (13.5%)	3 (6.7%)
Not Sure	--	2 (4.4%)
**Tumor Grade (n**,** %)**		
I	11 (11.5%)	4 (8.9%)
II	31 (32.3%)	7 (15.6%)
III	22 (17.7%)	7 (15.6%)
IV	17 (17.7%)	22 (48.9%)
Not Sure	15 (15.6%)	5 (11.1%)
**Tumor Hemisphere (n**,** %)**		
Left	41 (42.7%)	18 (40.0%)
Right	44 (45.8%)	20 (44.4%)
Bilateral	11 (11.5%)	7 (15.6%)
**Time Since Diagnosis (mean**,** range)**		
	71 months (0–389)	65 months (0–373)
**Treatment History (n**, **% not mutually exclusive)**		
Surgery	76 (79.2%)	37 (82.2%)
Radiation	66 (68.8%)	37 (82.2%)
Chemotherapy	60 (62.5%)	37 (82.2%)
**Psychological Variables**		
Depression (PHQ-9) (M, SD)	10.61 (6.79)	9.31 (6.08)
Generalized Anxiety (GAD-7) (M, SD)	8.43 (6.21)	9.84 (5.97)
Death Anxiety (DADDS) (M, SD)	29.44 (18.94)	39.27 (16.58)

### Patient PTG profile

70% of patients endorsed a total score above the cutoff for moderate-to-high PTG, with an *Appreciation of Life* being the most frequently endorsed subscale (68.8%). Nearly half of patients reported moderate-to-high PTG in the subscales *Relating to Others* (46.9%), *Spiritual Change* (40.6%), and *Personal Strength* (39.6%), with only 18.8% endorsing *New Possibilities* as an area of growth following their diagnosis (Table 2). Patients with high-grade classified tumors (grades III-IV; *M* = 60.0, *SD* = 19.3) reported higher PTG than patients with low-grade tumors (grade I-II; *M* = 51.2, *SD* = 17.1; *t* = -2.19, *p* = 0.03). Tumor type trended toward significance, *F*(3, 79) = 2.212, *p* = 0.09, with patients with glioblastoma (*M* = 65.0, *SD* = 20.3) reporting higher PTG than patients with meningioma (*M* = 48.0, *SD* = 24.9; *p* = 0.017), astrocytoma (*M* = 52.2, *SD* = 17.2; *p* = 0.066), or oligodendroglioma (*M* = 52.8, *SD* = 21.5, *p* = 0.063). PTG was positively associated with death anxiety (*r* = 0.272, *p* = 0.008). There was no association with age, time since diagnosis, depression, or generalized anxiety and PTG in patients (*ps* > 0.10). There was no difference in patient PTG between genders or tumor hemispheres (*ps* > 0.10).

### Caregiver PTG profile

Nearly half of caregivers scored above the cutoff for moderate-to-high PTG (48.8%), with *Appreciation of Life* subscale being the most frequently endorsed (53.3%), and the other subscales rated as moderate-to-high in about one-fourth to one-third of the caregiver sample (20–33%; Table 2). Despite not meeting statistical significance (*p* > 0.05), higher PTG was reported across most subscales in those caring for patients with high-grade classified tumors vs. low-grade tumors, except *Personal Strength* which was the only PTG subscale in which caregivers of those with low-grade tumors reported more frequent PTG. Tumor hemisphere trended towards significance, *F*(2, 42) = 3.087, *p* = 0.056, with caregivers of patients with left-hemisphere tumors (M = 56.9, SD = 27.1) reporting higher PTG than caregivers of patients with right-hemisphere tumors (*M* = 39.2, *SD* = 19.5; *p* = 0.020). There was no difference found in PTG between patients with bilateral tumors v. left- or right-hemisphere tumors (*ps* > 0.10). There was no association between PTG and age, time since diagnosis, depression, generalized anxiety, or death anxiety in caregivers (*ps* > 0.10). There was no difference in caregiver PTG between genders, relationship to patient, or patient tumor type.

### Comparison of patient and caregiver PTG

Differences between patient PTG and caregiver PTG trended towards significance, with patients reporting higher total PTG (M = 53.3, SD = 21.1) than caregivers (M = 46.7, SD = 23.6; *t* = -1.653, *p* = 0.10).

**Table 2:** PTGI profile and prevalence rates

	Patients		Caregivers	
	M (SD)	Moderate-to-High Qualifier	M (SD)	Moderate-to-High Qualifier
PTGI Total	53.29 (21.14)	70.1%	46.73 (21.62)	48.8%
PTGI Relating to Others	2.67 (1.16)	46.9%	2.67 (1.21)	26.7%
PTGI New Possibilities	2.00 (1.66)	18.8%	1.84 (1.19)	20.0%
PTGI Personal Strength	2.59 (1.65)	39.6%	2.27 (1.34)	33.3%
PTGI Spiritual Change	2.27 (1.78)	40.6%	1.73(1.78)	28.9%
PTGI Appreciation of Life	3.24 (1.21)	68.8%	3.03 (1.15)	53.3%

## Discussion

Post-traumatic growth (PTG) is a psychological construct that describes the positive transformations that may arise following a major life crisis, such as a diagnosis of a brain tumor. The current study describes the prevalence of PTG among patients with primary brain tumors and their caregivers, as well as identifies PTG associations with demographic, patient medical, and psychological variables. Our findings suggest that despite the hardship experienced following a brain tumor diagnosis, many can identify positive changes as a result of the disease, as over two-thirds of patients and approximately half of caregivers reported moderate-to-high PTG in our sample. Theories of meaning-making suggest that life-altering events may prompt individuals to search for meaning as a way to cope and adjust to stress [25]. During this process, individuals attempt to integrate their understanding of illness into their broader beliefs about the world and sense of self [26, 27]. Successful integration may allow individuals to reframe aspects of their illness experience in a more positive light, thereby facilitating PTG. Distinct features of a brain tumor diagnosis, including the high risk of recurrence, noncurative treatment options, psychological and existential distress, and progressive neurocognitive decline, may be particularly salient triggers for this meaning-making process and, in turn, PTG.

The overall pattern of PTG in our sample largely mirrors what has been described in previous neuro-oncology studies and across other cancer types: PTG was robust across age, gender, time since diagnosis, and tumor hemisphere [28, 29]. However, patients with higher-grade tumors, or more aggressive diagnoses (glioblastoma v. meningioma, astrocytoma, oligodendroglioma) reported higher PTG. This finding aligns with prior oncology research demonstrating greater PTG at more advanced stages of disease [13, 28, 30], potentially reflecting the role of sustained and elevated stress in catalyzing the meaning-making process. Lastly, consistent with the broader cancer literature, *Appreciation of Life* was the most highly endorsed PTG domain in the current study, whereas *New Possibilities* was less frequently endorsed [31–33]; this pattern may reflect the prognostic uncertainty and often life-limiting nature of a brain tumor diagnosis. Though few studies have examined clinical correlates of PTG, available evidence similarly suggests that time since diagnosis is not associated with PTG patterns [13], suggesting that PTG may reflect an active process in which intentional meaning-making, rather than the mere passage of time, is most influential.

The current study also found that caregivers reported less PTG than patients with brain tumors. This supports the growing body of research documenting greater distress and unmet psychosocial needs among caregivers relative to patients in this population [7, 8, 34]. Similar to patients, caregivers reported the highest PTG in the domain of *Appreciation of Life*, suggesting that the diagnosis may serve as an invitation to savor the remaining moments with their loved ones, and perhaps their own lives, as they are confronted by the inevitability of death. For caregivers, many aspects of PTG may emerge more fully following the patient’s death when caregivers have more emotional space to engage in the meaning-making process, given the substantial emotional burden and responsibilities experienced during active caregiving [35, 36]. Indeed, in a small sample of bereaved neuro-oncology caregivers, PTG was higher overall, with personal strength emerging as a particularly salient domain [37]. Caregiver PTG was similarly robust across age, gender, time since diagnosis, tumor type, and type of relationship to patient but generally higher among those caring for high-grade and left-hemisphere tumors. Because left-hemisphere tumors are related to poorer overall survival, as well as reduced cognitive and functional status [38, 39], caregivers of those with more deficits may come to appreciate the progressive decline of this disease earlier, thus triggering engagement in meaning-making processes that foster PTG. Nevertheless, future studies should replicate these findings in larger caregiver samples, and longitudinally, to better characterize the emergence and evolution of caregiver PTG across the illness trajectory and into bereavement.

The only identified psychological correlate of PTG among patients was elevated death anxiety. This may point to the tendency for existential fear to promote meaning-making and skillful coping behaviors in patients with cancer [40]. The co-occurrence between PTG and death anxiety may also point to double awareness – the ability to hold two seemingly opposing truths at once [41, 42]. For patients with brain cancer, the distress of a shortened future (death anxiety) may be present alongside a sense of meaning and appreciation of their lives (PTG). It is possible that PTG and death anxiety create a positive feedback loop wherein confrontation with death results in greater appreciation for life, which in turn contributes to a greater fear of losing that life. However, the cross-sectional nature of this study precludes our ability to understand the direction of these relationships and how these factors develop over time in this population. Additionally, we found no significant associations between PTG and symptoms of depression or anxiety. While the association between PTG and psychological symptomatology has not been assessed in previous research specific to neuro-oncology, findings from other cancer populations have been mixed [26, 43]. Taken together, these findings suggest that PTG represents a distinct and co-occurring psychological process rather than merely the absence of distress [42], underscoring the complexity of emotional adaptation to life-threatening illness.

### Limitations and future directions

This study provides important descriptive data on PTG among patients with primary brain tumors and their caregivers, including the first quantitative description of caregiver PTG and patients with both high- and low-grade tumors in neuro-oncology. Nevertheless, several limitations should be noted. Although the sample was heterogeneous with respect to tumor diagnosis, grade, and hemisphere, it was relatively homogeneous in terms of race and ethnicity, which may limit generalizability. In addition, patients and caregivers were permitted to enroll independently of one another, and disease characteristics could not be verified through the electronic health record, introducing the potential for error. Despite instructions to anchor PTG responses to the brain tumor diagnosis, participants may have experienced the COVID-19 pandemic and other unmeasured life events between diagnosis and data collection that influenced psychological outcomes. The benchmarks for categorizing the PTGI scores as low or moderate/high, while broadly used in the literature, warrant empirical investigation in cancer populations. Recruitment primarily from online support groups may have introduced selection bias, as participants who engage in these communities could differ systematically from those who do not. Most of our online recruitment strategy occurred through private, moderated groups requiring verification prior to access, which likely reduced any risk of feigning; participants were also not monetarily compensated for participation, further reducing incentive for fraudulent participation. Lastly, given the relatively small caregiving sample size and the number of statistical comparisons, future studies should seek to replicate these findings in larger, more diverse samples using longitudinal designs. Such studies would allow for examination of the temporal relationships between death anxiety and PTG—an important consideration for intervention development—and enable investigation of dyadic interdependence in PTG among patients and caregivers.

## Conclusion

A diagnosis of a primary brain tumor is often accompanied by profound emotional distress for both patients and their caregivers. Although the negative psychological sequelae of this diagnosis are well documented, positive psychological outcomes such as PTG remain comparatively understudied in neuro-oncology. In the present study, a substantial proportion of patients (70%) and nearly half of caregivers (48.8%) reported moderate-to-high levels of PTG, with appreciation of life emerging as the most prominent domain in both groups. Notably, PTG was higher in those with more aggressive tumors and positively associated with death anxiety, suggesting that psychotherapeutic approaches that explicitly engage with mortality and existential concerns may potentially facilitate PTG in this population. Together, these findings underscore the clinical relevance of PTG in neuro-oncology and highlight its potential to inform psychosocial, palliative, and end-of-life interventions for patients with primary brain tumors and their caregivers.

## Data Availability

The datasets generated during and/or analyzed during the current study are available from the corresponding author on reasonable request.
